# Therapeutic mechanisms of exclusive enteral nutrition in Crohn’s disease

**DOI:** 10.1007/s00281-025-01053-w

**Published:** 2025-07-02

**Authors:** Tina Krammel, Jiatong Nie, Deborah Häcker, Tobias Schwerd, Doriane Aguanno, Dirk Haller

**Affiliations:** 1 Chair of Nutrition and Immunology, Technical University of Munich, Gregor-Mendel-Strasse 2, 85354 Freising, Germany; 2 Department of Pediatrics, Dr. Von Hauner Children´S Hospital, University Hospital, LMU Munich, Munich, Germany; 3 ZIEL - Institute for Food & Health, Technical University of Munich, 85354 Freising, Germany

## Abstract

Crohn’s disease (CD) is a chronic, relapsing multifactorial inflammatory condition of the gastrointestinal tract, which is diagnosed under the age of 17 in 25% of patients, categorized as pediatric CD (pCD). Exclusive enteral nutrition (EEN) is a first-line therapy for inducing remission in pCD, yet its precise mechanisms remain poorly understood. This review summarizes the complex interplay of EEN-induced protective changes in the gut microbiota, epithelial barrier function and mucosal immune responses. EEN reshapes the gut microbiome by excluding potential pathobionts from the gut mucus layer and increasing protective bacterial and dietary metabolites. Emerging evidence highlights the role of EEN in modulating mitochondrial function, tryptophan metabolism and other metabolites in the intestinal epithelium and immune cells, which may contribute to its therapeutic efficacy. However, high variability in microbiome responses across clinical cohorts and discrepancies between clinical trials and animal models warrant further research to identify functional consequences and therapeutic mechanisms of EEN.

## Background of Crohn’s disease

Crohn’s disease (CD) is a chronic, relapsing inflammatory condition affecting the gastrointestinal tract (GIT) and is one of the leading entities of inflammatory bowel diseases (IBD) [1]. While ulcerative colitis (UC) is marked by a continuous inflammation limited to the mucosa and submucosa, specifically impacting the colon and rectum [2], CD is characterized by a patchy transmural inflammation affecting any region of the GIT. Twenty-five percent of patients with IBD are diagnosed under the age of 17, categorized as pediatric-onset IBD [3]. Pediatric CD (pCD) is often more severe, tends to progress more quickly and, compared to adult patients, affects a larger proportion of the GIT [4]. Furthermore, pCD patients face specific challenges, including growth impairment, delayed puberty and poor bone health [5]. Epidemiological studies show global variations in the prevalence and incidence of pediatric IBD, underscoring the complex factors involved in disease development [6]. While most industrialized countries have reached the stage of compounding prevalence, exhibiting stable incidence rates for CD, newly industrialized countries in Asia and South America are experiencing an increasing trend in disease incidence and prevalence (acceleration in incidence stage) [7, 8]. Additionally, the incidence of pCD, particularly very early-onset CD (VEO-CD), continues to rise globally [6]. Certain environmental factors (notably diet and gut microbiota) together with genetic susceptibilities contribute to the risk of developing CD. At present > 400 IBD risk loci have been identified across European and East Asian ancestries [9, 10] and include genes involved in host-microbiota interactions, highlighting the complexity of IBD etiology.

## CD pathogenesis involves changes in the gut microbiota, the epithelial function and the mucosal immune response

**Fig. 1:**
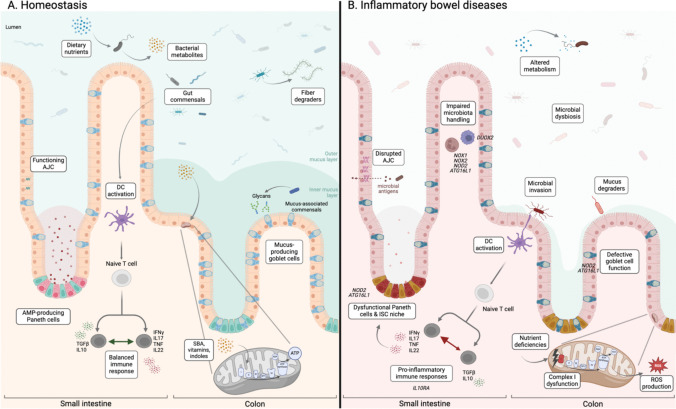
** Overview of function, metabolism and host-microbiota interactions and IBD-related changes in the small intestine and colon.** (**A**) Under homeostasis, the intestine is a functional complex barrier hindering microbes from reaching the epithelium and translocating through IECs, via the production of AMPs by Paneth cells (small intestine) and mucus (secreted by goblet cells) organized in two layers in the colon (inner sterile layer and an outer looser layer). Mucus and its constitutive glycans are also used as a source of nutrients by gut commensals. The latter are sensed by DCs and the balance between regulatory and pro-inflammatory immune processes guarantees appropriate immune responses from the lamina propria. AJC between adjacent cells maintain the monolayer of IECs cohesiveness and control the passage of molecules from the lumen, contributing to homeostasis. IECs take up nutrient-derived metabolites, like vitamins, fatty acids, BAs and indoles, which are important for mitochondrial homeostasis and ATP production. (**B**) In IBD, genetic risk variants (highlighted by gene names in italics) contribute to the observed impaired barrier function: dysfunction of Paneth cells (small intestine), reduced goblet cell numbers and overall reduced mucus thickness (colon) as well as impaired phagocytic and oxidative capacities of macrophages and neutrophils. Together with disrupted AJC, all these barrier defects contribute to an increased penetration of food- and bacterial-derived molecules, which trigger and perpetuate pro-inflammatory responses, also favoring the growth of inflammation-adapted microbial pathobionts. In return, the immune over-activation participates in IECs dysfunctions. Furthermore, in impaired mitochondria, a compromised nutrient metabolism, necessary for ATP production, results in the dysfunction of complex I of the ETC as well as in a release of ROS. IEC: intestinal epithelial cells, AMP: antimicrobial peptides, AJC: apical junctional complexes, BAs: bile acids, ATP: adenosine triphosphate, DCs: dendritic cells, IBD: inflammatory bowel disease, ETC: electron transport chain, ROS: reactive oxygen species, TGFβ: transforming growth factor β, Interleukin: IL, interferon-γ: IFNγ. Created in BioRender: https://BioRender.com/k88a961

The mucosal surface of the gut plays an essential role in providing barrier integrity and regulating immunological tolerance, with intestinal epithelial cells (IECs) mediating the homeostatic status between the gut microbiota and the host [11, 12]. IBD has been associated with a dysfunctional microbial community [13], a disrupted immune system [1] and an impaired mitochondrial function in IECs [14] (Fig. 1).

### CD-associated changes in epithelial barrier function and epithelial metabolism

IBD pathogenesis results from the complex interplay of environmental factors and genetic predispositions, rendering the intestine, and particularly the epithelium, more susceptible to a dysregulation of host-microbial dynamics. An intact barrier comprises a cellular, physiochemical and immunological component, which hinders intestinal microbes and toxins from entering the circulatory and lymphatic system. The first physical barrier is the mucus layer (produced by goblet cells), which contributes to the separation between microbiota and epithelium (Fig. 1). Nevertheless, the mucus is not an impenetrable barrier for all bacteria, even in a healthy environment, but acts as a habitat for the mucus-associated microorganisms, which attach to glycans and colonize the outer mucus layer [12]. Before their uptake or translocation across the gut interface, microbial metabolites, macromolecules and antigens reach the monolayer of IECs, sealed by tight junctions, which control the intestinal paracellular permeability (Fig. 1). An impaired intestinal barrier function, also known as a “leaky gut,” is observed in IBD, including a reduced number of mucin-producing goblet cells, disrupted tight junctions and compromised defensin production. These defects allow increased levels of luminal microbial- and food-derived antigens to penetrate the intestinal barrier [15] (Fig. 1). Bacteria infiltrating the mucus layer reach close proximity to the epithelial cell monolayer and are sensed by antigen-presenting dendritic cells (DCs), which then interact with immune cells to mount an immune response, including the production of B cell-derived secretory immunoglobulin A (sIgA) [16]. An increased bacterial penetration in the barrier, as seen in IBD, contributes to the excessive dysregulated immune response and perpetuation of chronic inflammation [17] (Fig. 1).

Noteworthy, various CD genetic susceptibilities, such as *NOD2*, *ATG16L1*, *IRGM*, *LRRK2* and *XBP1,* are involved in autophagy and endoplasmic reticulum (ER)-stress response, which are critical in handling microbiota through key IEC functions. Indeed, the synergy of the CD risk genes *NOD2* and *ATG16L1* lead to defective autophagy and antimicrobial response in Paneth cells [18, 19]. The role of autophagy, ER stress and *Nod2* has recently been expanded to goblet cell physiology and mucus secretion [20]. Overall, this highlights some shared features of Paneth and goblet cells as well as their respective defects and contributions to CD development [21].

CD pathogenesis is intrinsically linked to disrupted immune processes [1, 22]. Nonetheless, the intricate interaction of the epithelium with the underlying lamina propria affects IEC functions and control of the microbiota, in particular of pathobionts [23]. For example, loss of immunosuppressive responses as a result of Mendelian inheritance of the *IL10* and *IL10RA* gene variants leads to an impaired barrier function and the development of VEO-CD [24]. Noteworthy, mechanisms of colitis due to IL10R-deficiency involve pro-inflammatory macrophages, the activated Interleukin (IL) 23-IL22 axis, the accumulation of T helper 17 (Th17) and type 3 innate lymphoid cells (ILC3s) as well as IECs responses skewed toward antimicrobial activities [25, 26]. Interestingly, the development of VEO-IBD has particularly highlighted common cellular functions shared between immune and epithelial cells [27]. The impaired nicotinamide adenine dinucleotide phosphate (NADPH) oxidase capacity in phagocytes, neutrophils or IECs, through missense mutations in, respectively, *NOX2* [28], *NOX1* and *DUOX2* genes [29], has been identified as VEO-IBD risk factors and implicates defective oxidative responses. Similarly, NOD2 and ATG16L1 can promote phagocytic activities, through autophagy in immune cells such as macrophages, DCs and neutrophils and might contribute to CD pathogenesis, as already shown in Paneth and goblet cells. Consistently, we showed that Tumor necrosis factor (*TNF*)-mediated loss of stemness and subsequent development of Paneth cell dysfunction in the small intestine, in *Tnf*^*ΔARE/Wt*^ mice, contribute to the development of CD-like pathology [30], highlighting again the complex changes of epithelial function and handling of microbiota under inflammation.

In this context, the evidence to the role of metabolic alterations in IBD accumulates [31], in particular on the high-energy demand of the inflamed mucosa during healing processes, which have been long ago proposed under the “energy deficiency” hypothesis in UC [14]. Mitochondria are a cornerstone in intestinal homeostasis by controlling IEC metabolism, including oxidative phosphorylation (OXPHOS), and influencing IEC fate and properties [32]. Genes involved in mitochondrial function point toward its implication in IBD [33–35]. In line, morphological evidence of disruption of mitochondrial structure in IECs of IBD patients, including in non-inflamed areas [36–38], and impairment of mitochondrial genes and function in the mucosa of active UC have been shown at both transcriptional [39] and protein levels [40, 41]. Consistently, we demonstrated that the functional plasticity of the intestinal epithelium and its regenerative response to injury are controlled by mitochondrial metabolism [42, 43] and its crosstalk with the microbial milieu [44]. Furthermore, we showed that mitochondrial dysfunction in the intestinal stem cell (ISC) niche, which leads to an aberrant Paneth cell fate, predicts disease recurrence in CD patients [30], emphasizing its importance in the regenerative capacity of the intestinal epithelium. Under normal physiological conditions, the differentiated epithelium performs mitochondrial respiration to consume oxygen for ATP production, resulting in physiological tissue hypoxia and anaerobic conditions in the intestinal lumen [45]. Under inflammatory conditions and impaired mitochondrial function, the lack of oxygen consumption by IECs and the increased mitochondrial production of reactive oxygen species (ROS) and nitrogen species (RNS) from infiltrating phagocytes favor the growth of facultative anaerobes in the intestinal milieu, thus contributing to the loss of intestinal homeostasis [45]. Elevated ROS production due to a dysfunction of the electron transport chain (ETC) in IECs can exacerbate disease progression [46]. Respiratory measurements of IBD patient biopsies from the active inflammation site showed a reduced complex I activity of the ETC. Further analysis revealed a reduced membrane potential as well as a reduction in PGC1α, an important regulator of mitochondrial biogenesis [39]. Further research has underscored the importance of mitochondria in various metabolic pathways, including fatty acid oxidation [47] and amino acid metabolism. In pCD patient samples, acylcarnitines are enriched in their feces, suggesting less cellular or mitochondrial uptake due to mitochondrial dysfunction in those patients [48]. Malnutrition and micronutrient deficiencies, which include important substrates for functioning mitochondria, are associated with IBD [49].

Overall, these various changes in the intestinal epithelium in CD highlight the complex interactions between host IECs, immune cells and gut microbiota.

### CD-associated dysbiosis of the gut microbiota

The gut microbiota, composed of trillions of indigenous microorganisms, is a crucial component of the intestinal ecosystem, exerting a profound influence on both gut and systemic health by shaping and maintaining gut barrier integrity, immune tolerance and metabolic homeostasis [50–52]. Disease-associated changes of the gut microbiota, often referred to as dysbiosis, are suggested to contribute to IBD pathogenesis. In addition to a reduced microbial diversity in CD patients (Fig. 1), the dysbiotic shifts in the microbiota involve an increased abundance of Enterobacteriaceae family members [13, 23] as well as mucin-degrading *Ruminococcus gnavus* and *R. torques*, which possess polysaccharide utilization loci [12, 53]. Increased abundances of *Fusobacterium*, *Streptococcus*, *Bacteroides* and *Phocaeicola* spp. is also frequently reported [44, 54–56]. In addition, IBD-related mouse models identified colitogenic potential of some pathobionts, which can adhere to and invade the host epithelial barrier. These include segmented filamentous bacteria (SFB), *Helicobacter hepaticus* and *Klebsiella pneumonia,* which are associated with a Th1 or Th17-mediated pro-inflammatory response [57–59]. *Enterococcus faecalis* was also found to be colitogenic in mono-associated *Il10*^*−/−*^ and *Tnf*^*ΔARE/Wt*^ mice, due to the production of a metalloprotease GelE [60]. In summary, genome-wide association studies [61], clinical interventions [62, 63] and experimental evidence in gnotobiotic animal models [64] strongly support the hypothesis that the microbial intestinal milieu contributes to the development of chronic intestinal inflammation, explicitly in CD.

### CD-associated changes of bacterial- and diet-derived metabolites on immune responses

Short-chain fatty acids (SCFAs), including acetate, propionate and butyrate, are bacterial metabolites from fiber fermentation. They play an important role in maintaining intestinal homeostasis by enhancing tight junctions, stimulating mucus secretion and promoting an anti-inflammatory response [65]. In healthy individuals, butyrate, as a primary energy substrate of colonocytes, is taken up by the colonic epithelium via monocarboxylate transporters (MCT), including MCT1, MCT4 and MCT5. It is used as a substrate for β-oxidation and mitochondrial respiration, the main route of ATP production in colonocytes [47]. SCFAs can also activate anti-inflammatory signaling cascades by targeting G-protein coupled receptors (GPCRs) such as GPR41, GPR43 and GPR109A on immune cells and IECs [66]. They also upregulate IL22 production by promoting the aryl hydrocarbon receptor (Ahr) and hypoxia-inducible factor 1α (HIF1α) expression [67]. Compared to healthy populations, CD patients exhibit reduced intestinal levels of SCFAs and SCFA-producers such as *Clostridium*, *Roseburia intestinalis* and *Fecalibacterium prausnitzii* [68].

IBD has been associated with an increase in primary bile acids (PBAs), such as cholic acid, and a decrease in secondary bile acids (SBAs), such as lithocholic acid (LCA). Both, the malabsorption and changes in the gut microbiota, which normally convert PBAs to SBAs, can be linked to the BA imbalance [23, 69, 70] (Fig. 1). The fecal metabolome from pCD patients is also characterized by decreased levels of unconjugated BAs and SBAs, which is accompanied by reduced BA-metabolizing bacteria such as *Eubacterium* and *Ruminococcus* [71]. Metabolomic, metagenomic and transcriptomic profiling of stool from UC patient pouches shows a reduced abundance of LCA and deoxycholic acid (DCA), reduced levels of SBA-producing Ruminococcaceae, as well as overall bacterial genes required to convert PBAs to SBAs [69]. Certain *E. lenta* strains were found to express either the 3α hydroxysteroid dehydrogenase (*hsdh*) or the 3β *hsdh*, which are necessary enzymes to convert LCA into 3-oxoLCA or isoLCA [72]. Those secondary plant metabolites and the *E. lenta hsdh* homologs are significantly depleted in inflamed samples from CD and UC patients compared to their healthy control tissue [72].

Taken together, the changes in all compartments and actors of intestinal homeostasis observed in CD highlight the intricate complexity of its physiopathology and the challenge to understand its mechanisms for therapeutic interventions.

## Mechanisms of exclusive enteral nutrition (EEN) as a dietary treatment for pCD

Dietary exposure plays an important role in IBD pathogenesis by directly influencing the gut mucosal immune responses or altering the gut microbiome, collectively interacting with host allostasis [73]. A number of dietary therapies, such as EEN, the Mediterranean diet and Crohn's Disease Exclusion Diet (CDED), have been studied in large cohorts or randomized clinical trials (RCTs) in the past decades to show their clinical efficacy of inducing remission or controlling disease activities in IBD patients [3, 74–77]. Among these, EEN remains to be the primary treatment for inducing remission in pCD recommended by the European Society of Pediatric Gastroenterology, Hepatology and Nutrition and the European Crohn's and Colitis Organization due to its high efficacy in inducing remission (67–80% of patients), the ability to improve nutritional status, promote growth, and its favorable safety profile [78, 79]. EEN has demonstrated comparable efficacy to corticosteroids and has also shown clinical efficacy in adult patients [79, 80]. Notably, it is superior to corticosteroids in promoting mucosal healing, which is a predictor of long-term sustained remission following EEN therapy [81–84]. EEN comprises low-fiber or fiber-free liquid formulas based on either milk protein, predominantly casein and whey protein (polymeric diet), or single amino acids (elemental diet). Despite varying protein compositions and other differences, median remission rates among these formulas are not significantly different [81, 85, 86].

### Clinical efficacy of EEN and its impact on the gut microbiota and host response

Numerous clinical trials investigated the influence of EEN on microbial abundance, changes in dietary and bacterial metabolites, as well as the host immune response. A summary of these studies is provided in Table 1. CD-associated dysbiosis is characterized by a reduced microbial diversity, an increased abundance of Proteobacteria (e.g., *E. coli*) and a reduced abundance of Bacillota (previously known as Firmicutes, e.g., *F. prausnitzii*) [87, 88]. While EEN has demonstrated clinical efficacy [89, 90], it has been associated with a further reduction in microbial richness and diversity in some studies, though these findings are not consistent across all studies [89, 91–93]. Notably, certain presumably beneficial bacteria, which already exhibit a reduced abundance in CD patients, such as *F. prausnitzii* and *Bifidobacterium,* decline further during EEN treatment in several studies [83, 89, 90, 94]. The discussion of these paradoxical evidence such as increased fecal pH and change of fecal hydrogen sulfide (H_2_S) levels or H_2_S producers during EEN is still ongoing [89, 95, 96]. Despite some overlapping findings, the impact of EEN on microbiota also varies widely among clinical studies. Indeed, the microbiome of CD patients is highly heterogeneous, both inter-individually and intra-individually, across the disease course. Our recent longitudinal pCD cohort also demonstrates substantial variations and dynamic changes of the gut microbiome at strain level, between and within individuals longitudinally in response to the EEN treatment. In addition, responders and non-responders to EEN therapy exhibit distinct microbiota and metabolome profiles at baseline. Responders experience normalization of markers with EEN, showing slightly increased BA metabolism and reduced microbial diversity [92]. Lv et al. suggest that an increased level of Bacillota and secondary unconjugated BA may contribute to the remission of CD [71]. Elevated levels of trimethylamine and cadaverine, which hinder epithelial growth and adherence, are observed in CD but normalized with EEN. These metabolites may play a role in EEN-induced remission by modulating lipopolysaccharide-stimulated cytokine secretion in primary human lymphocytes [92]. In addition, patients who achieve sustained remission after EEN treatment harbor more predominant strains from *Akkermansia muciniphila* and Bacteroides and are limited in Proteobacteria, which is more prevalent in patients relapsing at early time points [97]. Besides the different changes in the gut microbiome and metabolome in responder and non-responder patients, carriers of the *NOD2* frameshift mutation obtained the greatest benefit in response to EEN. In contrast, specific *NOD2* genotypes are associated with higher relapse rates [98]. Therefore, the question of whether distinct bacterial taxa can be directly linked to EEN-induced remission remains unresolved and requires further evaluation.

**Table 1:** Summary of clinical findings with EEN interventions and microbiome/metabolome analysis in pediatric CD patients

Study population and design	Dietary intervention and remission rate	Changes in gut microbiota, dietary, & bacterial metabolites	Changes in host immune response
Mild to moderate pCD patients (Prospective RCT) [3]	CDED + PEN (*n* = 40) vs. EEN (*n* = 34) in two phases (6 weeks each); 75% corticosteroid-free remission in CDED + PEN group and 59% corticosteroid-free remission in EEN group after 1 st phase	W6 and W12 remission from both diet groups: decreased *Haemophilus*, *Veillonella*, *Bifidobacterium*, *Prevotella*, and *Anaerostipes*, and increased *Oscillibacter* and *Roseburia* EEN group: decreased *Lachnospira* and increased *Subdoligranulum*, *Blautia*, *Ruminococcus*, and *Erysipelotrichaceae*	NA
Mild to moderate pCD patients (Prospective RCT): [75] (substudy of the above trial)	CDED + PEN (*n* = 17 on W6) vs. EEN (*n* = 14 on W6) in two phases (6 weeks each); 88% clinical remission in CDED + PEN group vs. 71% in EEN group after 1 st phase	CDED + PEN W6 remission: decreased kynurenine & succinate synthesis; increased *N*-α-acetyl-arginine EEN W6 remission: changes in lipid metabolism EEN compared to CDED + PEN: decreased purine, pyrimidine, & sphingolipid metabolic pathways in fecal metabolome	NA
Mild to moderate pCD patients (Prospective RCT): [99] (substudy of the first trial)	CDED + PEN (*n* = 22) vs. EEN (*n* = 21) in two phases (6 weeks each); 87% clinical remission in CDED + PEN group vs. 69% in EEN group after 1 st phase	CDED + PEN- & EEN- induced W6 and W12 remission: reduction of kynurenine & quinolinic acid from the kynurenine pathway W6 remission: increased melatonin & indole W12 remission: increased melatonin & 5-OH-tryptophan Biomarker signatures of EEN-induced remission: reduced kynurenine/melatonin & quinolinic acid/melatonin	NA
Active pCD patients (Prospective cohort): [100]	8 weeks of EEN (*n* = 66); 62% clinical remission	NA	EEN remission patients: decreased plasma concentration of IL6, IL17E, IL17F & IL31; significant positive correlation between fecal calprotectin & IFNy, IL1β, IL6, IL8, IL17a, IL17F, IL22, IL23, IL27, IL31 & IL33
Newly diagnosed pCD patients vs. healthy controls (Prospective cohort): [71]	8 weeks of EEN (*n* = 27) vs. healthy controls (HC; *n* = 27); 92% clinical remission	CD compared to HC at baseline: increased relative abundance of Proteobacteria & reduced Bacteroidetes; Increased primary unconjugated BA Post-EEN compared to pre-EEN: increased Shannon diversity & relative abundance of phylum Bacillota; Increased levels of genera *Clostridium V* & *Flavonifractor.* Primary unconjugated BA was similar to HC; Remission patients had increased levels of secondary BA	NA
Newly diagnosed pCD patients with active disease (RCT): [83]	8 weeks of EEN (*n* = 13) *vs.* 4 weeks of steroids (*n* = 6); 100% clinical remission in EEN group *vs*. 83% in steroid group. 89% mucosal healing of EEN group *vs*. 17% in steroid group	Post-EEN compared to pre-EEN: Shannon’s index increased; Increased relative abundance of species of *Clostridium XIVa* & *IV. C. symbiosum*, *C. ruminantium, R. torques, R. gnavus* & *Clostridium hathewayi* Steroid treatment: enriched *Roseburia intestinalis, Eubacterium* & *Bifidobacterium bifidum*	EEN group compared to steroid group: reduced levels of IL17 & IFN within the intestinal mucosa; no difference in cytokine profiles
Active pCD patient vs. healthy controls (Prospective cohort): [89]	8 weeks of EEN (*n* = 15) vs. healthy controls (*n* = 21); 80% clinical remission	During or Post-EEN compared to pre-EEN: bacterial richness decreased significantly; reduced *F. prausnitzii* spp & *Bacteroides/Prevotella*; Higher fecal pH & total sulfide; lower butyric acid Returning to normal diet compared to post-EEN: increased *F. prausnitzii* spp & *Bifidobacterium* genus; *Bacteroides/Prevotella* returned to pretreatment level; reduced proportion of D-lactate	NA
Active pCD patients disease vs. healthy controls (Prospective cohort): [90]	8 weeks of EEN (*n* = 23) vs. healthy controls (*n* = 21); 62% clinical remission	CD compared to HC at baseline: reduced Shannon diversity; reduced *Bifidobacterium adolescentis*, *Ruminococcus bromii*, *Eubacterium* spp*.*, *F. prausnitzii*, *Coprococcus eutactus*, & *Subdoligranulum variabile*; increased *Streptococcus anginosus*, *Enterococcus faecalis*, & *R. gnavus* & *Clostridium clostridioformes* Post-EEN compared to pre-EEN: Further reduced Shannon diversity; increased *Lactococcus*; reduced *Bifidobacterium, Dialister, Ruminoccocus* & *Faecalibacterium;* reduced abundance of genes involved in biotin & thiamine biosynthesis but increase in those involved in spermidine/putrescine biosynthesis	NA
Active pCD patients (Prospective cohort): [91]	6–8 weeks of EEN (*n* = 15)	Post-EEN compared to pre-EEN: Shannon effective diversity not affected; increased relative abundance of Bacillota (family Ruminococcaceae and Christensenellaceae); decreased relative abundance of Bacteroidetes (family Bacteroidaceae, Porphyromonadaceae, Rikenellaceae)	EEN treatment: increased relative and absolute numbers of FOXP3 + Treg cells in the peripheral blood; reduced Treg cells in the lamina propria of patient tissue biopsies *In vitro*: isolated PBMCs during EEN stimulated with LPS or flagellin secreted less inflammatory cytokines IL-6, IL-8, IL1β, & TH1-derived IFNγ but not TNF or IL17; EEN enhanced capacity of IL10 to suppress LPS-induced IL6 in PBMCs
pCD patients vs. healthy controls (Prospective cohort): [94]	8 weeks of EEN (*n* = 12) vs. healthy controls (*n* = 16); 100% clinical remission	CD compared to HC at baseline: Reduced Shannon index; enrichment of genera *Escherichia, Peptostreptococcus* & *Morganella;* reduced abundance of species *Anaerostipes hadrus*, *Blautia wexlerae*, *F. prausnitzii*, *B. uniformis*, *Eubacterium hallii*, *R. bromii*, *Fusicatenibacter saccharivorans*, *B. longum*, & *B. pseudocatenulatum*; decreased SBAs & unconjugated BAs; decreased levels of HDCA, βHDCA, apoCA, EDCA, αMCA, dehydroLCA, NorDCA, TLCA, LCA, isoLCA & DCA Post-EEN compared to pre-EEN: Increased genera *Hungatella*, *Parvimonas*, *Clostridioides*, *Solobacterium*, *Clostridium*, & *Enterococcus*. Increased species *Actinomyces sp oral taxon 414*, *C. innocuum*, *C. perfringens*, *Clostridioides difficile*, *Hungatella hathewayi*, *Parvimonas micra*, & *Solobacterium* moorei; decreased *B. stercoris*, *Haemophilus parainfluenzae*, & *Veillonella atypica*; increased levels of SBA and unconjugated BAs; increased levels of HCA, αMCA, & 6-keto-lithocholic acid	*In vitro*: HCA suppressed TNFα production and IFNγ- and TNFα- producing cells in the CD4 + T cell population using PBMCs isolated from CD patients
Newly diagnosed pCD patients [101]	6–8 weeks of EEN (*n* = 20); 90% clinical remission	EEN compared to post EEN group: increased *E. bolteae, C. innocuum, C. symbiosum, Thomasclavelia ramosa, C. scindens, Ruthenibacterium lactatiformans, Hungatella hominis, E. coli, Eisenbergiella tayi, R. torques* & *Hungatella hatheway*i. *F. duncaniae* & *Anaerostipes hadrus* etc. are negatively associated with EEN compared to post EEN samples. Increased fecal levels of palmitoleic acid, decanoic acid, caprylic acid, & lauric acid during EEN	NA

### Impact of EEN on the modulation of gut microbiota and metabolites in mouse models

**Fig. 2:**
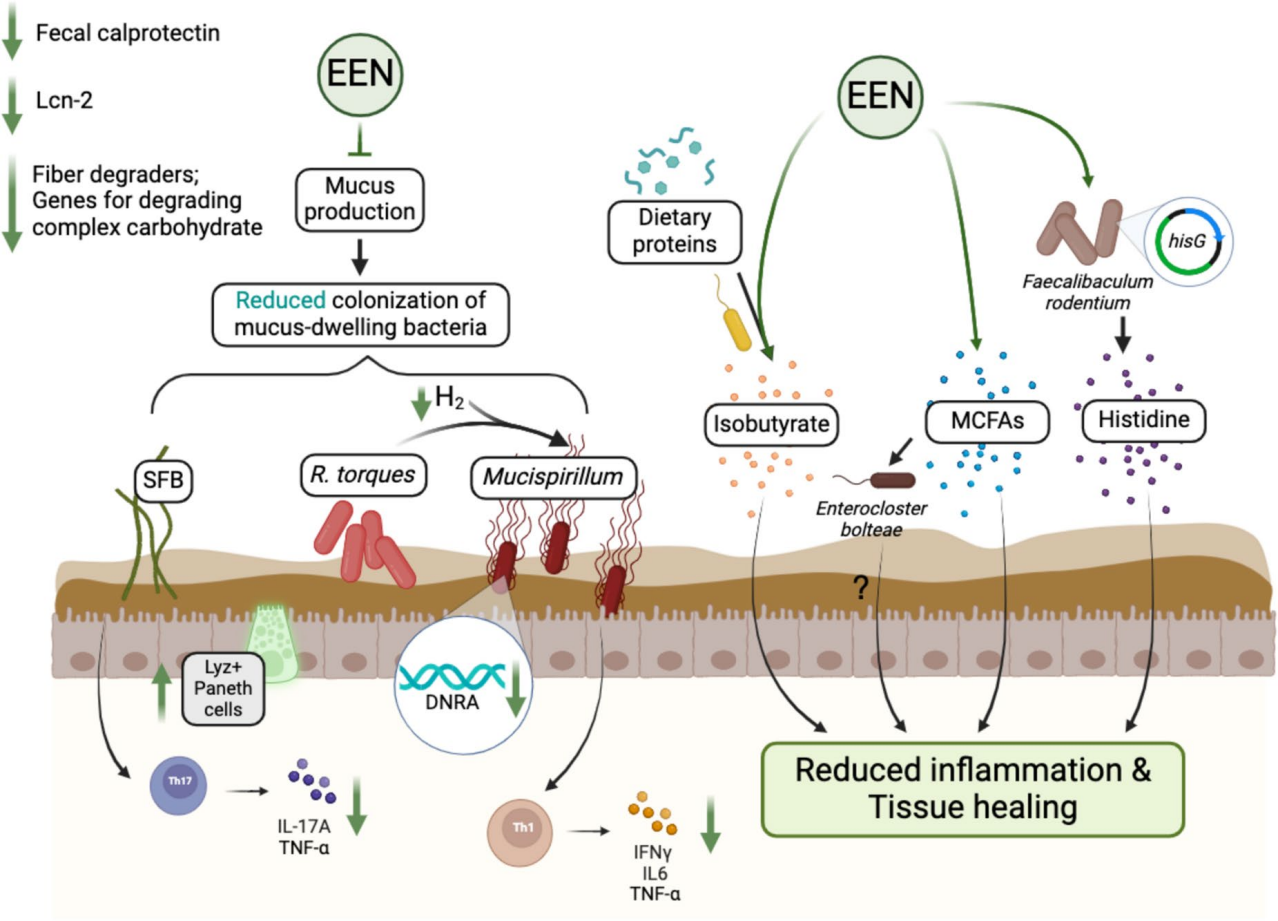
**Putative protective mechanisms of EEN through the modulation of gut microbiota and metabolites in mouse studies.** An EEN-like diet resolves inflammation in the distal ileum of the *Tnf*^*ΔARE/Wt*^ mouse model by preventing the colonization of the pathobiont SFB in the mucus layer. This leads to an increase in lysozyme-positive Paneth cells and a reduction in inflammatory cytokine production. In the colon of a double-knockout Nod2 and Cybb mouse model, an EEN-like diet reduces fermentative hydrogen (H_2_) production by *Ruminococcus torques*. This limits H_2_ availability for *Mucispirillum*, thereby suppressing its dissimilatory nitrate reduction to ammonia (DNRA) pathway and reducing its colonization in the mucus layer, along with inflammatory cytokine production. EEN also increases the bacterial metabolite isobutyrate, derived from protein fermentation, which alleviates cecal inflammation in *Il10*^*−/−*^ mice. Additionally, EEN enhances the abundance of *Faecalibaculum rodentium*, which produces histidine and mitigates colonic inflammation in *Il10*^*−/−*^ mice. Changes in dietary fatty acid profiles, such as increased MCFAs, may also contribute to reduced inflammation via microbiota-dependent or independent mechanisms. SFB: segmented filamentous bacteria, MCFAs: medium-chain fatty acids, DNRA: dissimilatory nitrate reduction to ammonia, Lcn-2: Lipocalin-2. Created in BioRender: https://BioRender.com/k88a961

A few recent *in vivo* studies have explored the effect of EEN-like diets as a dietary intervention on mouse models of chronic intestinal inflammation. The inflammatory conditions of pCD can be replicated in *Il10*^*−/−*^ mice through fecal microbiota transfer (FMT) from pCD patients at various disease stages. Notably, the *ex-vivo* cultivated gut microbiota from an active disease state in an EEN-like media protects against inflammation in *Il10*^*−/−*^ mice, suggesting a diet-induced functional change of patient-derived gut microbiota, independent of the host [101]. Additionally, medium-chain fatty acids (MCFAs), supposedly originating from IBD Modulen®, are identified as signature metabolites associated with EEN treatment in a longitudinal cohort of pCD patients. These MCFAs are able to selectively activate a certain EEN-response (e.g. *Enterocloster* spp. and *Escherichia coli*), underscoring the potential of EEN to reshape gut microbiota composition and function through targeted interactions with dietary metabolites [101] (Fig. 2).

Experimental evidence suggests that EEN-induced remission can be mediated by the exclusion of potentially pathogenic bacteria from the host’s mucus layer. Mono-colonization with SFB in *Tnf*^*ΔARE/Wt*^ mice causes CD-like inflammation. The growth and colonization of SFB is prevented by an EEN-like purified diet devoid of fibers, leading to a reduced inflammatory phenotype in the mouse model of ileitis. On the other hand, the growth of SFB is supported by a control chow diet, which results in a reduced number of lysozyme-positive Paneth cells, elevated expression of *Tnf* and *Il17a*, leading to ileocolonic inflammation in these mice [102] (Fig. 2). An EEN-like fiber-free diet also inhibits the spontaneous development of CD-like colitis in mice with a double knockout of two CD susceptibility genes, *Nod2* and *Cybb* [103]. In this study, the fiber-free diet alters the localization of a disease-causing bacterium, *Mucispirillum,* whose colonization relies on the mucus layer. *Mucispirillum’s* growth is promoted by hydrogen (H_2_) production from the mucin degrader *R. torques*, a taxon closely related to species from the Lachnospiraceae family. This family has shown reduced abundance during fiber deprivation [103] (Fig. 2).

Specific bacterial metabolites play an essential role in mediating the protective effects of EEN. Zeng et al. demonstrated enriched *Faecalibaculum rodentium* in *Il10*^*−/−*^ mice, which received liquid enteral nutrition treatment. Gavaging either *F. rodentium* or the supernatant of this bacterial culture alleviates colonic inflammation in *Il10*^*−/−*^ mice. The increased bacterial synthesis of histidine, encoded by the *hisG* gene, is responsible for this protective effect [104]. Dietary histidine also demonstrates anti-inflammatory properties in macrophages of the *Il10*^*−/−*^ cell transfer colitis model [105]. Additionally, a protein-derived bacterial metabolite isobutyrate is also found to be enriched and protective against cecal inflammation in *Il10*^*−/−*^ mice colonized by a synthetic consortium composed of 14 bacterial strains and fed a pelletized commercial EEN diet [106]. Both isobutyrate and butyrate could equally offset the diet- and microbiome-induced inflammation in these colonized *Il10*^*−/−*^ mice [106]. In line with this observation, higher isobutyrate concentration is also identified in *Il10*^*−/−*^ mice receiving an EEN-conditioned microbiota and an EEN-like diet [101]. The mechanistic role of isobutyrate is not clearly understood yet, but the increased levels of branched-chain fatty acids during EEN treatment may function as an alternative energy source in colonocytes under conditions of low butyrate availability [107].

The fiber paradox in the IBD context is an intriguing topic. In contrast to the acknowledged health benefits of dietary fibers, the majority of EEN formulas are devoid of any fibers [85]. While EEN feeding reduces inflammation in *Il10*^*−/−*^ mice, the fiber-free nature of this diet reduces mucus thickness, potentially due to the expansion of mucin degraders such as *Akkermansia muciniphila* and *B. caccae* [106]. Clinical studies present contradictory outcomes regarding disease flare-ups upon fiber consumption in CD patients with active disease [108–110]. During food reintroduction after EEN in pCD, higher levels of fecal calprotectin are linked to a higher intake of fiber, gluten-containing cereals and processed red meat despite higher fecal butyrate levels observed in this group [111]. In fact, the potential adverse effects of dietary fiber in IBD patients suggest that intestinal inflammation may result from the absence of fiber-degrading bacteria in the microbiota [112].

Similar to EEN, a diet low in fermentable oligosaccharides, disaccharides, monosaccharides and polyols (FODMAPs) demonstrates clinical efficacies for relieving gastrointestinal symptoms and improving stool consistency in irritable bowel syndrome and IBD patients in the post-acute phase [113–115]. A low FODMAP diet also diminishes fecal abundance of SCFA-producers such as *Bifidobacterium adolescentis*, *Bifidobacterium longum* and *Faecalibacterium prausnitzii*, which is likely due to the reduced level of colonic fermentable substrates [116]. Similarily, the CD-TREAT low-fiber whole-food-based diet was designed to mimic the dietary composition of EEN while excluding potential triggers of intolerance, with clinical efficacy in a small trial study involving pCD patients. Both EEN and CD-TREAT could induce comparable changes in fecal microbiome composition and metabolome (e.g. reduced SCFAs) in healthy adult participants [117]. Despite the previously described necessity of butyrate in mitochondrial and cellular metabolism [47, 65], a reduction in SCFAs and a normalization of metabolites related to protein degradation is reported in EEN-responders [96]. Moreover, EEN leads to a reduction of gut bacterial enzymes (e.g., glycoside hydrolases) that are responsible for degrading complex plant carbohydrates such as arabinoxylans and pectic polysaccharides and an increase in enzymes responsible for the digestion of simple sugars such as sucrose or short fructooligosaccharides. Furthermore, genes involved in butyrate production decrease in the EEN group compared to a vegan or omnivore group in a healthy population [118].

Taken together, experimental data underscore the notion that the therapeutic effectiveness of EEN acts through alterations in the gut microbiota and specific dietary and bacterial metabolites. However, despite unraveling mechanisms by using animal models under well-controlled dietary interventions, the selective contribution of distinct bacterial taxa from murine microbiota fails to explain the protective mechanisms of EEN in patients. For example, SFBs are absent in mucosal biopsies in adult and pediatric IBD patients [102]. Due to the fiber-free nature of most EEN formulas, multiple clinical studies observe increased levels of mucolytic bacteria and reduced levels of fiber-degrading bacteria (Fig. 2). Contradicting the experimental evidence in mice [103], mucin degraders, such as *R. torques* and *R. gnavus*, are found to be significantly increased during EEN treatment in human subjects [83, 118, 119]. Therefore, shifts in microbiota composition due to EEN are insufficiently translated into functional targets of disease pathogenesis. The partly contradictory results regarding host inflammatory response to dietary fiber also need further evaluation in the context of IBD.

### Impact of EEN and EEN-induced changes of bacterial metabolites on intestinal epithelial function

**Fig. 3:**
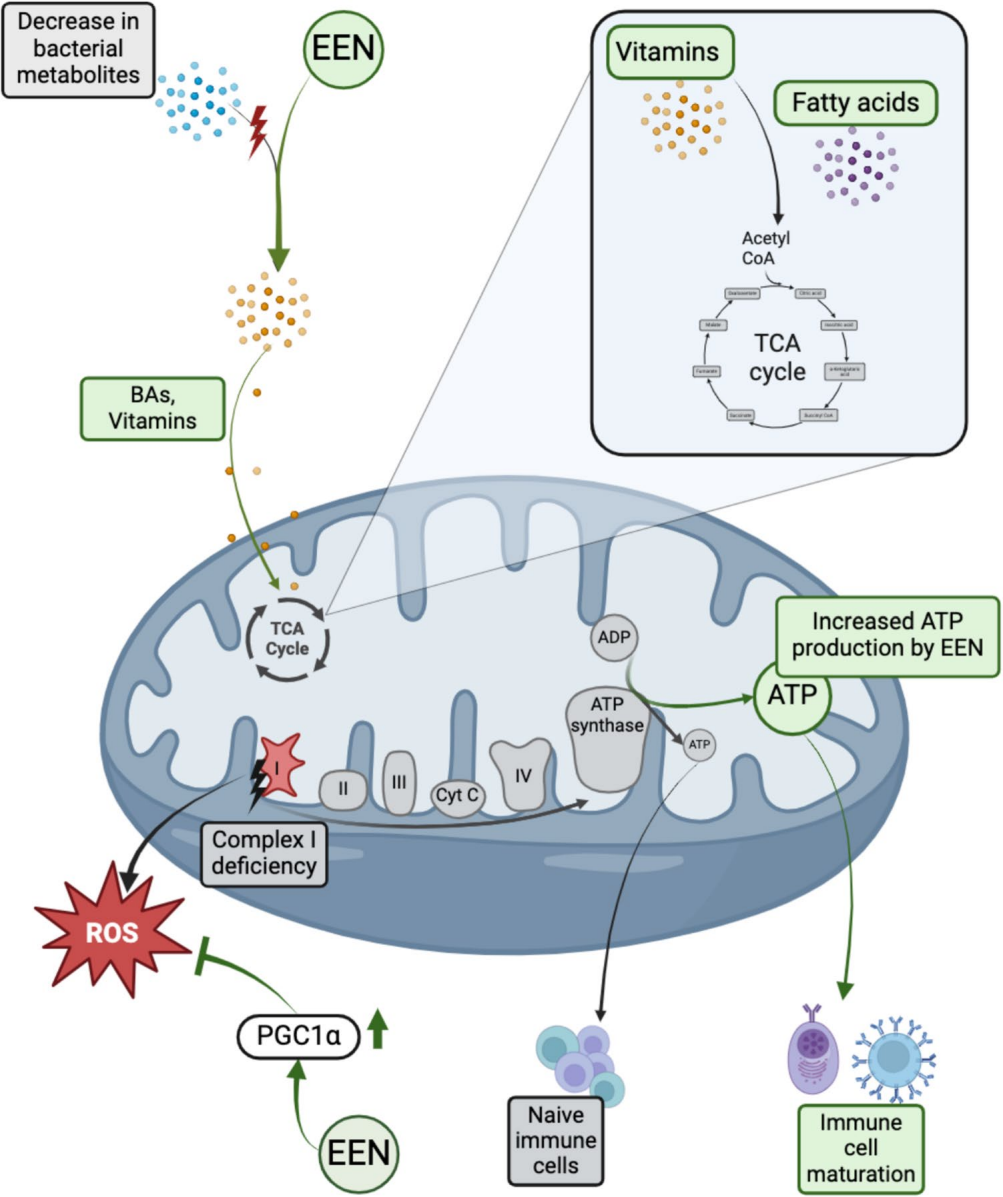
**Mitochondrial biogenesis in an inflammatory system with a display of functional enhancements potentially due to EEN treatment.** In an IBD situation, the microbial changes lead to a reduction in the availability of bacterial metabolites like fatty acids, bile acids (BAs) - especially secondary BAs - and Vitamins, which dampens mitochondrial activity and energy production. An increase in or supplementation of vitamins and fatty acids is associated with EEN treatment, which can feed into the tricarboxylic acid (TCA) cycle and subsequently enhance ATP production. Additionally, EEN treatment enhances the PGC1α expression, inhibiting ROS production due to a complex I deficiency in IBD. The overall increase in ATP production is necessary not only for the mitochondrial biogenesis, but also for immune cell maturation and the release of anti-inflammatory signals. Created in BioRender: https://BioRender.com/k88a961

Restrictive diets coupled with malabsorption and other clinical complications can lead to malnutrition in IBD [120]. The fecal metabolome indicates a depletion of vitamins and fatty acid-related molecules in CD compared to healthy controls [121]. Vitamin B9 (folate) and vitamin B12 deficiencies are common among CD patients, especially among those who have undergone ileal or colonic resections. Both vitamins are integral to the one-carbon (1C) metabolism, which plays a crucial role in synthesizing amino acids, DNA and the regulation of the methionine cycle [122]. In this context, EEN therapy enhances lean body mass and increases plasma levels of these micronutrients (e.g. vitamins) with antioxidant properties [123], which can play a crucial role in safeguarding against mitochondrial toxicity and oxidative stress [124, 125] (Fig. 3). Malnourished IBD patients have also demonstrated reduced mitochondrial complex I activity compared to healthy subjects [126], as well as a reduced membrane potential with a reduction in PGC1α, an important regulator of mitochondrial biogenesis [39]. One week of nutritional support, including enteral nutrition, is able to significantly increase mitochondrial complex I activity of peripheral blood mononuclear cells (PBMC) from IBD patients [126] (Fig. 3). Some clinical trials recently started to investigate mitochondrial danger-associated molecular patterns as a biomarker to evaluate the inflammation grade or apply mitochondrial antioxidant therapy in pCD patients in order to assess the effects of this dietary supplementation on mitochondrial damage and ROS production (ClinicalTrials.gov identifiers: NCT04760964 and NCT05539625).

**Fig. 4:**
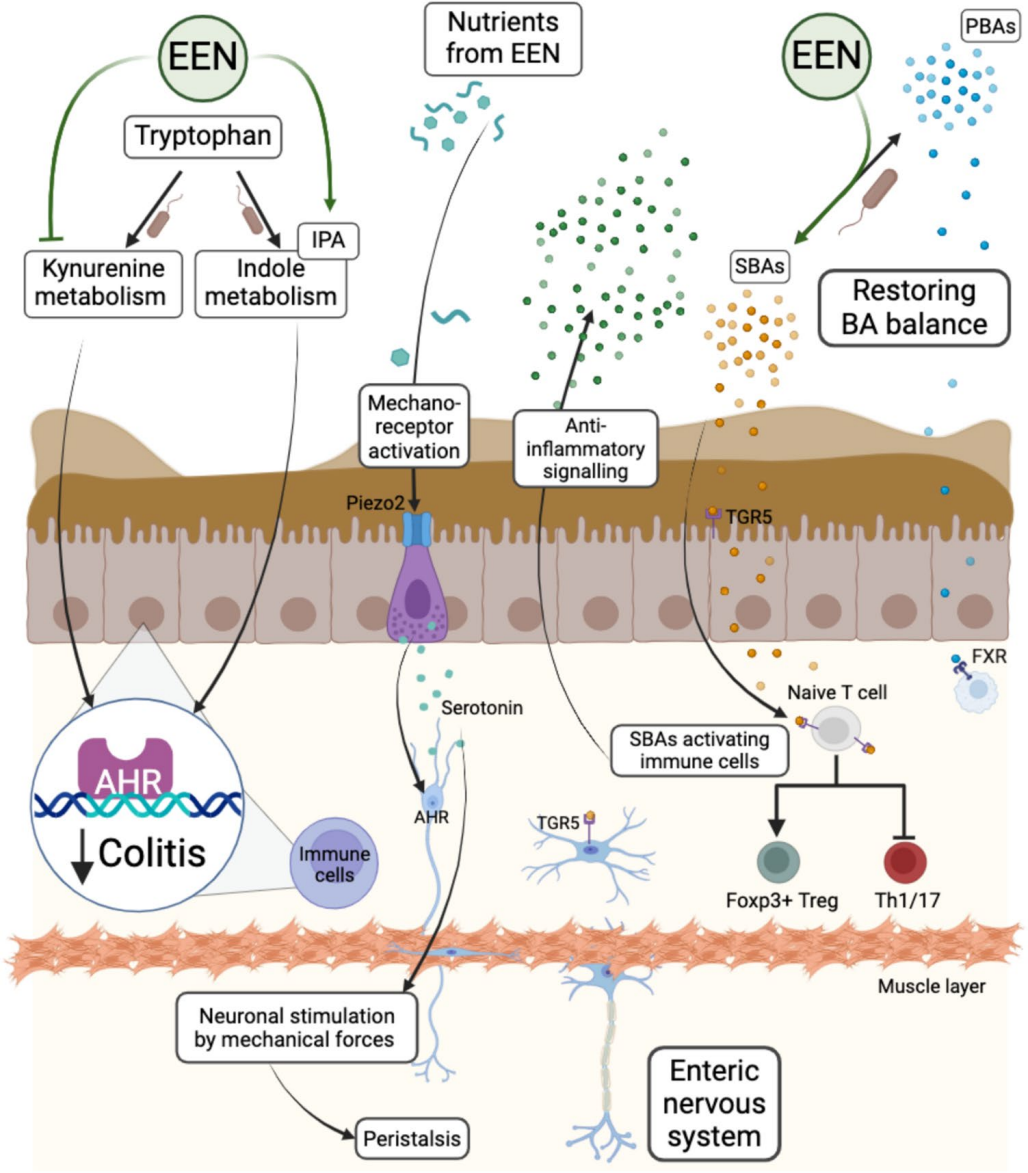
**Display of the tryptophan and bile acid metabolism as well as the regulation of intestinal peristalsis.** EEN treatment shifts the tryptophan metabolism leading to a reduced kynurenine and an increased indole production. Indoles act as ligands for the AHR receptor on immune cells, alleviating colitis severity. Indoles can also activate the AHR of enteric neurons, influencing the intestinal peristalsis. Additionally, the stimulation of mechanoreceptors like Piezo2 on enteroendocrine cells leads to the release of serotonin, which activates the AHR receptor on enteric neurons, also influencing peristalsis and thus transit time. Furthermore, EEN restores the balance of SBAs and PBAs by increasing the number of bacteria that can metabolize PBAs. The BAs bind to specific receptors, like TGR5 and FXR, which inhibit the differentiation of Th17 immune cells while also enhancing the differentiation of regulatory T cells, thus promoting an anti-inflammatory signalling. AHR: aryl hydrocarbon receptor, SBAs/PBAs: secondary bile acids/primary bile acids, TGR5: Takeda G-protein coupled receptor, FXR: farnesoid X receptor. Created in BioRender: https://BioRender.com/k88a961

Severe and chronic inflammation in CD leads to significant tissue abnormalities, such as the formation of edema and fibrotic changes. These alterations can result in partial obstruction and increased mechanical stress due to the accumulation of food. Mechanical stress results in enhanced Th1 and Th17 immune responses [127], thereby exacerbating inflammation [128]. One important mechanosensor triggering the release of serotonin in the gut is the calcium-permeable mechanosensitive ion channel Piezo2. A subpopulation of enteroendocrine cells expressing Piezo2, are electrically excitable and react to a broad range of stimuli, like nutrients and metabolites, but also mechanical forces, and thus were shown to influence intestinal motility and food transit time [129] (Fig. 4). EEN as a liquid diet enhances the transit time by reducing mechanical stress and activating mechanoreceptors [127, 129]. In a rat model of colitis, mechanical stress is present at the site of inflammation and proximal to it, leading to increased production of pro-inflammatory cytokines IL6 and Osteopontin (OPN). Feeding liquid EEN instead of solid food reduces food accumulation in the intestine. This decrease in luminal content alleviates intestinal distention, relieves mechanical stress and attenuates mechanotranscription (the transcriptional regulation of mechanosensitive genes) (Fig. 4). Additionally, it reduces levels of IL6 and OPN and attenuates Th17 response in the mechanically distended inflammation site [127]. However, the CDED + PEN diet is as effective as EEN but allows patients to consume certain solid foods, while eliminating dietary components, which supposedly contribute negatively to the microbiome. Nevertheless, roughly 50% of the daily consumed calories in the first six weeks are covered by the liquid enteral nutrition diet [3], which supports the mechanical stress theory.

Enteric neurons surrounding the intestine also play a role in regulating the intestinal peristalsis (Fig. 4), particularly through Ahr, whose expression increases with the bacterial load along the GIT. Dietary and microbial ligands of Ahr, like indoles derived from tryptophan metabolism, lead to the activation of feedback regulators or regulators of the neuronal excitability affecting the peristalsis in the colon [130]. Ahr activation by bacterial tryptophan metabolites decreases the severity of inflammation in experimental models [47], while a reduction in tryptophan serum levels in combination with an elevation in the kynurenine pathway activation is associated with active CD [131]. A significant reduction of kynurenine and quinolinic acid, as well as an increase in melatonin is associated with both CDED + PEN (partial enteral nutrition) and EEN-induced remission in a 12-week prospective randomized trial in pCD with mild to moderate disease activity, suggesting a diet-induced alteration of the kynurenine and serotonin pathway [75, 99]. Indoles are produced through bacterial tryptophan metabolism and are important regulators to sustain barrier function as well as an anti-inflammatory response [132]. However, a metabolite milieu low in bacteria-derived indoles is reported as a result of EEN therapy and associated with remission in various studies [99, 118]. This may be explained by the influence of fermentable fiber availability on tryptophan metabolism. A fiber-free diet favors indole production, whereas a fiber-rich diet promotes the production of indole acetic acid and indole propionic acid. This difference is due to cross-feeding interactions between fiber-degrading bacteria and those with gene expressions for tryptophan metabolism [133] (Fig. 4).

In clinical trials, EEN improves the reabsorption of BAs in the small intestine and restores the microbial balance, including bacteria, which convert PBAs to SBAs [71]. EEN restores the balance of PBA and SBAs in the gut of responder pCD patients by increasing levels of SBAs [71, 94] (Fig. 4). It additionally leads to enriched levels of hyocholic acid (HCA), which *in vitro* reduces IFNγ- and TNF-producing cells in the CD4 + T cell population using PBMCs isolated from CD patients. The EEN-induced increase in SBAs is accompanied by an increased abundance of BA-metabolizing bacteria such as *Clostridium inocuum* and *Hungatella hathewayi* [94]. The balance of BAs and subsequent activation of their receptors, like the farnesoid X receptor (FXR) for PBAs, are important for the integrity of the intestinal barrier and are impaired in CD patients. Furthermore, the secondary BA LCA is the primary activator of the Takeda G protein-coupled receptor 5 (TGR5) on ileal and colonic epithelial cells and promotes epithelial barrier regeneration [71, 134] (Fig. 4).

These described protective effects of EEN on diverse epithelial functions and metabolism, with a microbial crosstalk, highlight the multifaceted impact of this therapy and the need to better discriminate its therapeutic actions on immune responses and microbial changes at the mechanistic level.

### Effect of EEN-induced changes of bacterial and dietary metabolites on immune modulation

The previously described EEN-mediated increase in SBA production is not only important for the cellular integrity and barrier functions, but also for their immunomodulatory effects [135, 136]. In general, naïve T cells can differentiate into pro-inflammatory cells (Th1 or Th17 cells) or anti-inflammatory cells (Foxp3^+^, IL10 + regulatory T cells) [137, 138]. The restoration of SBAs with EEN treatment leads to the induction of anti-inflammatory effects (reduced IL6, TNFα, increased IL10) by binding of SBAs to target receptors (FXR and G protein-coupled receptor 1) on monocytes, macrophages and dendritic cells [136] (Fig. 4). Moreover, the two derivatives of LCA (3-oxoLCA or isoalloLCA) display T-cell regulatory actions. 3-oxoLCA inhibits Th17 cell differentiation, while isoalloLCA influences the production of mitochondrial ROS, thus enhancing regulatory T-cell differentiation [135]. EEN therapy also increases FOXP3^+^ regulatory T cells (Tregs) in the peripheral blood, while reducing Tregs in the lamina propria, which is thought to be a consequence of the resolution of mucosal inflammation [91]. Proteomics has enabled identification of key proteins involved in immune modulation during EEN treatment. A recent study described an alteration of inflammation-related proteins predominantly in EEN-treated patients who also showed a decrease in fecal calprotectin. Those patients displayed decreased levels of plasma proteins, such as MMP-1, IL6 and CCL23, which play a role in the tissue remodeling, neutrophil or Th17 pathway. Additionally, EEN treatment enhanced immune pathways involved in intestinal physiology (fibroblast growth factor 19 and 23), as well as pathways in inflammation control (Il10 and Il10RB) [139].

Part of the dietary metabolite alterations are changes of fatty acid profiles in the gut due to EEN treatment, which may also interact with the immune system. EEN treatment enhances changes in lipid metabolism, particularly affecting linoleic acid and sphingolipids metabolism, which leads to immune response modulation and reprogramming by regulating T-cell activation and CD8 + T-cell function [75]. Besides the previously described interaction of MCFAs with certain gut bacteria in mediating the effect of EEN, the elevated level of MCFAs in the fecal metabolome of pCD patients consuming IBD Modulen® [101] can also exert anti-inflammatory properties by reducing the expression of TLR-4 and pro-inflammatory cytokines and improving barrier function by increasing the intestinal stem cell differentiation and enhancing expression of tight junction proteins [140, 141]. Nevertheless, EEN formulas containing various fatty acid compositions also lead to disease remission accompanied by changes of fecal fatty acids [85, 142]. This warrants further investigations of their interactions with the gut immune system and cellular functions to understand their potential therapeutic benefits.

Meanwhile, increased levels of antibodies, like IgG, can arise as a response to food antigens (e.g. soybean, wheat, corn) [143]. CD patients have a higher seroreactivity to a wide range of food items compared to healthy subjects [143]. Experimental data suggests that levels of serum IgG antibodies against food proteins are higher in the *Il10*^*−/−*^ colitis model than in WT mice [143]. EEN feeding in TNBS-induced colitis mice reduced IgA and IgG-coated bacteria in their feces compared to chow-fed mice [144]. Therefore, EEN may exert its therapeutic effects by reducing immune reactivity to food antigens and gut microbiota.

## Conclusion

EEN, as the first-line therapy of pCD, has demonstrated protective mechanisms by modulating different components of the gut ecosystem (gut microbiota, epithelial barrier functions and mucosal immune response). However, the effects of EEN on specific bacterial populations are largely variable across clinical cohorts and some pathobionts discovered in animal models lack clinical relevance. The search for microbial signatures that could serve as specific biomarkers to predict the response to EEN therapy in CD also remains incomplete [145, 146]. This highlights the need to identify the functional relevance of alterations in certain microbes and metabolites induced by EEN. Further research into alternative diets—such as CD-TREAT, the Specific Carbohydrate Diet, CDED and the Mediterranean Diet—is essential, given their better patient tolerance and comparable clinical outcomes [74, 76, 117]. Ultimately, closing the knowledge gaps around how EEN influences host cellular functions, including mitochondrial metabolism and immune signaling through microbiota modulations, is the key to advancing targeted, microbiome-based therapies for sustaining remission.

## Data Availability

No new data were generated or analysed in this review.
