# Correction: Therapeutic mechanisms of exclusive enteral nutrition in crohn’s disease

**DOI:** 10.1007/s00281-025-01067-4

**Published:** 2025-11-11

**Authors:** Tina Krammel, Jiatong Nie, Deborah Häcker, Tobias Schwerd, Doriane Aguanno, Dirk Haller

**Affiliations:** 1 Chair of Nutrition and Immunology, Technical University of Munich, Gregor-Mendel- Strasse 2, 85354 Freising, Germany; 2 Department of Pediatrics, Dr. Von Hauner Children´S Hospital, University Hospital, LMU Munich, Munich, Germany; 3 ZIEL - Institute for Food & Health, Technical University of Munich, 85354 Freising, Germany

**Correction: Seminars in Immunopathology (2025) 47:28**

**https://doi.org/10.1007/s00281-025-01053-w**

 In the original article first names of all authors were displayed as family names and family names as first names.

 The original article has been corrected.

